# A Reverse Mutation E143K within the PrM Protein of Zika Virus Asian Lineage Natal RGN Strain Increases Infectivity and Cytopathicity

**DOI:** 10.3390/v14071572

**Published:** 2022-07-20

**Authors:** Chen-Sheng Lin, Wei-Jing Li, Chih-Yi Liao, Ju-Ying Kan, Szu-Hao Kung, Su-Hua Huang, Hsueh-Chou Lai, Cheng-Wen Lin

**Affiliations:** 1 Division of Gastroenterology, Kuang Tien General Hospital, No. 117, Shatian Rd, Shalu District, Taichung 433, Taiwan; b8401126@yahoo.com.tw; 2 Department of Medical Laboratory Science and Biotechnology, China Medical University, No. 100, Sec. 1, Jingmao Rd., Beitun Dist., Taichung 404, Taiwan; jolin9367@gmail.com (W.-J.L.); angelliaoj302@gmail.com (C.-Y.L.); 3 The PhD Program of Biotechnology and Biomedical Industry, China Medical University, No. 100, Sec. 1, Jingmao Rd., Beitun Dist., Taichung 404, Taiwan; s0975782938@gmail.com; 4 Department of Biotechnology and Laboratory Science in Medicine, National Yang Ming Chiao Tung University, Taipei 112304, Taiwan; szkung@nycu.edu.tw; 5 Department of Medical Laboratory Science and Biotechnology, Asia University, No. 500, Lioufeng Rd., Wufeng, Taichung 41354, Taiwan; shhuang@asia.edu.tw; 6 Division of Hepato-Gastroenterology, Department of Internal Medicine, China Medical University Hospital, No. 2, Yude Rd., North Dist., Taichung 404, Taiwan; t674233@ms54.hinet.net

**Keywords:** Zika virus, Asian lineage, prM, amino acid substitution, single round infectious particle (SRIP), infectious clone (i.c.), infectivity, cytopathicity

## Abstract

Zika virus (ZIKV) is a positive-sense single-stranded RNA virus in the Flaviviridae, which is classified into two different lineages Asian and African. The outbreak of ZIKV Asian lineage isolates in 2015–2016 is associated with the increase in cases with prenatal microcephaly and Guillain–Barré syndrome, and has sparked attention throughout the world. Genome sequence alignment and the analysis of Asian and African lineage isolates indicate that amino acid changes, particular in positively charged amino acid substitutions in the pr region of prM protein might involve a phenotypic change that links with the global outbreak of ZIKV Asian-lineage. The study generated and characterized the virological properties of wild type and mutants of single-round infectious particles (SRIPs) and infectious clones (i.c.s) of ZIKV Asian-lineage Natal RGN strain, and then identified the function of amino acid substitutions at the positions 139 [Asn139→Ser139 (N139S)] and 143 [Glu143→Lys143 (E143K)] in ZIKV polyproteins (located within the pr region of prM protein) in the infectivity and cytopathogenicity. The E143K SRIP and i.c. of Natal RGN strain exhibited relatively higher levels of cytopathic effect, EGFP reporter, viral RNA and protein synthesis, and virus yield in three types of human cell lines, TE617, SF268 and HMC3, compared to wild type (WT), N139S SRIPs and i.c.s, which displayed more efficiency in replication kinetics. Additionally, E143K Natal RGN i.c. had greater activities of virus attachment and entry, yielded higher titers of intracellular and extracellular virions, and assembled the E proteins near to the plasma membrane in infected cells than the other i.c.s. The results indicate that the positively charged amino acid residue Lys143, a conserved residue in the pr region of prM of ZIKV African lineages, plays a crucial role in viral replication kinetics, including viral attachment, entry, assembly and egress. Thus, the negatively charged amino acid residue Glu143 within the pr region of prM leads to an alteration of the phenotypes, in particular, a lower replication efficiency of ZIKV Asian-lineage isolates with the attenuation of infectivity and cytopathicity.

## 1. Introduction

Zika virus (ZIKV) is a mosquito-borne flavivirus of the family *Flavivirus* that was first discovered in monkeys of the Zika forest in Uganda in 1947 and in *Aedes africanus* mosquitoes in 1948. ZIKV is a small, enveloped virion containing a single-strand, positive-sense RNA genome of nearly 11 kb [1]. Flavivirus genome has a large open reading frame (ORF) and two noncoding regions at 5′ and 3′ ends. Its ORF is translated as a big fusion polyprotein that is divided into structural (capsid (C), membrane (prM/M), envelope (E) proteins) and non-structural (NS1, NS2A, NS2B, NS3, NS4A, NS4B, NS5) proteins through the proteolytic process by viral proteases (NS2B-NS3) and host cell proteases. The E protein is the surface protein for the binding to the receptor and fusion to the membrane of the cells. NS3 is a multi-domain protein with the N-terminal protease (NS3pro), C-terminal RNA triphosphatase and helicase. NS5 consists of N-terminal methyltransferase and C-terminal RNA polymerase. ZIKV spreads from Africa to Southeast Asia through the transmission by *Aedes* mosquitoes, such as *Ae. albopictus*, *Ae. aegypti* and *Ae. Polynesiensis* and exhibits a genetic variation with the classification of African and Asian lineages [2]. ZIKV causes fewer than 20 human cases with self-limiting mild illnesses, such as a low-grade fever, headaches, rash, myalgia, arthralgia, and conjunctivitis reported from the 1960s to 1980s [3]. Recently, the ZIKV Asian-lineage (ZIKV Asian) strains spread from Southeast Asia toward the Pacific, leading to occur three large outbreaks in the Yap Island (Micronesia) in 2007, French Polynesia in 2013–2014, Brazil in 2015 and then across the Americas in 2016. The cases of the microcephaly in infants and Guillain–Barré syndrome (GBS) in adults significantly increased during the Zika outbreaks in French Polynesia and Brazil [4]. Human-to-human (sexual and vertical) transmission of ZIKV Asian-lineage strains was reported, as associated with the ZIKV persistence in blood and body fluids (tears, saliva, semen, cervical mucus and urine) [5]. Therefore, the WHO Emergency Committee announced ZIKV as a “Public Health Emergency of International Concern” (PHEIC) in November 2016 [6]. Nowadays, epidemic ZIKV Asian-lineage strains have been transmitted worldwide; laboratory-confirmed symptomatic cases of ZIKV infection are still reported [7]. The emergence of ZIKV is an extraordinary and enduring challenge to public health, revealing the importance of the objectives of the proposal in understanding the relationship between genetic evolution and the virulence of the epidemic ZIKV Asian-lineage strains.

The epidemic ZIKV Asian-lineage strains show a broad tropism in various tissues, for which the permissive cell types of ZIKV infection include brain cells, ocular cells, the reproductive tract cells, placenta cells, and endothelial cells [6,8]. Among these permissive cells of ZIKV infection, the brain cells, placenta cells, and endothelial cells are associated with the congenital ZKIV microcephaly. In in vitro assays, the epidemic ZIKV Asian-lineage strains display slower replication kinetics with lower peak virus titers and vRNA synthesis in human neural precursor cells (hNPCs)/human neural stem cells (hNSCs), human monocyte derived-dendritic cells (DCs), primitive human placental trophoblast, and other vertebrate cells than ZIKV African-lineage strains and pre-epidemic ZIKV Asian-lineage strains [9,10,11]. Interestingly, the epidemic ZIKV Asian-lineage strains form markedly larger plaques on plaque assays with human umbilical vein endothelial cells (HUVECs), showing faster rates of viral RNA synthesis in HUVECs and inducing a stronger cell death of HUVECs than the ZIKV African-lineage strains [8]. Therefore, the epidemic ZIKV Asian-lineage strains have differ in their cell tropism, allowing the intrauterine transmission from mother to fetus.

Phylogenetic analyses of ZIKV stains from 1947 to 2017 in Africa, Asia and America indicate that that some mutations gathered in the ZIKV epidemic strains. ZIKV Asian-lineage epidemic strains show 23 polymorphisms in comparison with the Micronesia strain and 5 polymorphisms within NS1 (K940E, T1027A, and M1143V), NS4B (T2509I) and NS5 (M2634V) compared to French Polynesia isolate [12]. An N-linked glycosylation site in the residue Asn-154 l of the E protein appears in ZIKV Asian-lineage strains, involved in mosquito-cell infectivity and virus assembly [13]. In addition, a single Alanine-to-Valine substitution (A983V) in the NS1 protein of ZIKV Asian-lineage pre-epidemic strain leads to antagonize IFN-β activation by inhibiting TBK1 phosphorylation [14]. The genetic changes in ZIKV Asian-lineage epidemic strains might be associated with the virulence, neurotropism, and transmissibility of ZIKV Asian-lineage strains. Importantly, the single Serine-to-Asparagine substitution (S139N) within the prM protein of ZIKV Asian-lineage pre-epidemic strains that was detected in the 2013 outbreak strains in French Polynesia markedly intensifies the neurovirulence with the infectivity to neural progenitor cells and the induction of severe microcephaly [15]. Recently, a chimera ZIKV African-lineage MR766 replaced with the prM gene of Asian genotype PRVABC59 isolate was shown to be less virulent compared to wild type MR766 virus in IFNAR-/- mice [16]. Thus, the amino acid changes in the prM protein might be involved in the phenotypic change, particularly in the attenuation of viral virulence that causes the global outbreak of ZIKV Asian-lineage.

**Figure 1:**
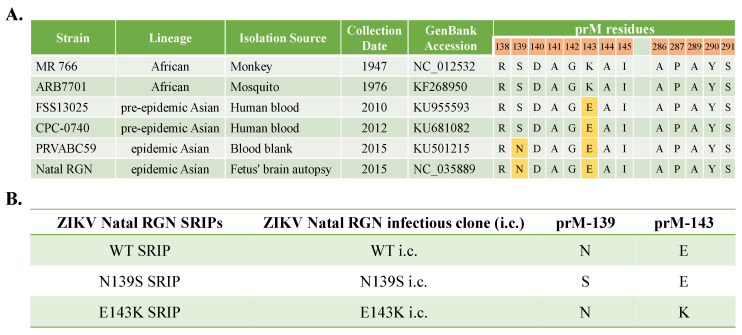
Amino acid alignment analysis of pre-epidemic African and epidemic ZIKV strains (**A**) and the name list of wild type, mutant single-round infectious particles (SRIPs) and infectious clones (i.c.s) of recombinant ZIKV Natal RGN (**B**).

To gain insights into the molecular determinants involved in the attenuation of viral virulence of ZIKV Asian-lineage, a comparative genomic analysis of pre-epidemic African-lineage strains and epidemic Asian-lineage strains revealed that 7 amino acid substitutions occurs in the precursor (pr) region of M protein (prM), implying that these genetic mutations could play a critical role in the disease phenotypes. Among the amino acid substitutions within the pr region of M protein (prM) (Figure 1A), the charged amino acid substitution of lysine-143 in the ZIKV African lineage replaced by glutamic acid-143 in ZIKV Asian lineage [Lys143→Glu143 (K143E)] was suggested to play the role in the phenotypic change in ZIKV Asian-lineage epidemic isolates in this study. The single-round infectious particle (SRIP) of the ZIKV Asian-lineage Natal RGN strain was generated using synthetic and reverse genetic technologies in our laboratory [17]. The ZIKV Asian-lineage Natal RGN strain is detected in brain tissues from fetal autopsy cases with microcephaly in the Natal region of Brazil; its genome was sequenced using next-generation sequencing (GenBank accession number KU527068) [18]. The ZIKV Natal RGN strain that is associated with microcephaly was recognized as the representative, epidemic, ZIKV Asian-lineage strain in 2015–2016. The study investigated the relationship between phenotypic change and reverse mutations in the pr region of prM in recombinant ZIKV. Wild type (WT) and mutant (N139S, E143K) SRIPs and infectious clones (i.c.s) of ZIKV Natal RGN strain were generated using reverse genetic approaches (Figure 1B), further characterizing their infectivity and cytopathicity, and their replication kinetics were examined, in particular, virus attachment, entry, assembly and egress in human rhabdomyosarcoma, human glioblastoma cells, and human fetal brain-derived primary microglia. 

## 2. Materials and Methods

### 2.1. Cells

TE671 (human rhabdomyosarcoma), SF268 (human glioblastoma), HMC3 (human fetal brain-derived primary microglia), and BHK-21 (baby hamster kidney fibroblast) cells were cultured in minimum essential medium containing 10% fetal bovine serum, 2 mM glutamine, 1 mM pyruvate, and 1× penicillin-streptomycin at 37 °C with 5% CO_2_. TE671 and SF268 cells were used for establishing the cell lines expressing ZIKV prM, prM-N139S, or prM-E143K plus wild type E protein, and then generating wild type (WT), N139S, and E143K SRIPs of ZIKV Natal RGN. Meanwhile, BHK-21 cells were utilized to produce prM-WT, prM-N139S, and prM-E143K infectious clones (i.c.s) of ZIKV Natal RGN, as well as to determine the virus titer of wild type and mutant SRIPs and i.c.s of ZIKV Natal RGN. In addition, TE671, SF268, and HMC3 cells were exploited to examine the biological properties of wild type and mutant SRIPs and i.c.s of ZIKV Natal RGN.

### 2.2. Generation of Single Round Infectious Particles (SRIPs) of ZIKV Asian-Lineage Natal RGN Strain with a Single Reverse Mutation within PrM Protein

To clone the reverse mutation at Residues 139 and 143 within the prM protein, the mutant prM-E gene was amplified using PCR with the mutagenic primers (Supplemental Figure S1A, bottom) and the template of wild type prM-E gene from the ZIKV Natal RGN genome (GenBank accession number KU527068), as described in our previous report [17]. The PCR products of mutant prM-E gene were digested with EcoRI and XhoI, and then cloned into the expression plasmid pcDNA3.1-HisC (Supplemental Figure S1A). The recombinant plasmids prM-WT/E, prM-N139S/E, and prM-E143K/E, were sequenced to confirm the desired mutations within the prM gene, respectively (Supplemental Figure S1B). The resultant plasmids were subsequently transfected with TE671 cells at 90% confluence in a 6-well plate with Lipofectamine LTX (Invitrogen, Carlsbad, CA, USA), according to the manufacturer’s guidelines. The transfected cells were selected in the culture media with 500 µg/mL G418 for 2 weeks, stably expressed wild type or mutant prM plus E proteins, which were named as prM-WT/E, prM-N139S/E, and prM-E143K/E cell lines, respectively. The expression of prM and E proteins was validated by real-time RT-PCR with E-specific primer pair and immunofluorescence staining with primary antibodies against ZIKV prM protein (GeneTex, Inc., Hsinchu City, Taiwan) plus secondary AF546 goat anti-rabbit IgG (Thermo Fisher Scientific, Waltham, MA, USA). To generate wild type and mutant SRIPs of ZIKV Natal RGN strain (GenBank: KU527068), each cell line was further transfected with a DNA-launched CMV promoter-driven replicon of ZIKV Natal RGN strain containing an enhanced green fluorescent protein (EGFP) reporter, constructed and characterized in our prior report [17]. The cytopathic effect (CPE) and EGFP expression in mock and replicon-transfected cells were inspected 24, 48, and 72 h post transfection. The yield and antigenicity of WT, N139S, and E143K SRIPs in the cultured media of replicon-transfected cells were assessed using real-time RT-PCR with NS5-specific primer pair, median tissue culture infectious dose (TCID50) assay in BHK-21 cells, and Western blotting with anti-E specific antibodies. In a TCID50 assay, serial dilutions of the stock were added and incubated on the 90% confluent monolayer of prM/E-expressing cells in 96-well plates. After incubating for 72 h at 37 °C, the CPE in each well was observed and recorded to determine the TCID50 titer of each stock of wild type and prM-mutant SRIPs. To analyze the antigenicity of ZIKV SRIPs, 75 μL of each SRIP stock was mixed with 25 μL of 4X Loading Dye, boiled for 10 min, and then loaded in 10% SDS-PAGE for running the gel electrophoresis. After electrophoretically transferring total proteins onto the nitrocellulose membrane, the immune-reactive bands were reacted with anti-E antibodies (GeneTex, Inc.) and HRP-conjugated anti-mouse IgG antibodies (Invitrogen, Carlsbad, CA, USA), detected by ECLTM Western Blotting Detection Reagents (GE Healthcare, Chicago, IL, USA), and then imaged by the Multi-function Gel Image System (MultiGel-21) (Gentaur, San Jose, CA, USA).

### 2.3. Generation of Wild Type and PrM-Mutant Infectious Clones of ZIKV Natal RGN Strain

To produce wild type and prM-mutant infectious clones (i.c.s), a DNA-launched CMV promoter-driven replicon of ZIKV Natal RGN strain containing a unique NotI restriction enzyme site between FMDV-2A (F-2A) and E’ (from ZIKV Nucleotide 2307) of ZIKV Natal RGN was digested by NotI restriction enzyme, and then in vitro ligated with the PCR production of prM-WT/E, prM-N139S/E, and prM-E13K/E using Gibson Assembly reaction (New England Biolabs, Ipswich, MA, USA) (Supplemental Figure S2). Wild type and mutant prM-E gene fragments (Residues 123–727) were amplified using PCR assay with the templates of the recombinant plasmids (prM-WT/E, prM-N139S/E, and prM-E143K/E), receptively (Supplemental Figure S2B). The PCR product was cloned into the NotI-digested ZIKV Natal RGN replicon using Gibson Assembly reaction (Supplemental Figure S2C). FMDV-2A (F-2A)-prM-E (Residues 123–727)-(Ala728-Ala729-Ala730)-E’(Residue 731~) was cloned via in-frame gene fusion into ZIKV Natal RGN replicon (Supplemental Figure S2A). Each mixture from the Gibson Assembly reaction of wild type or mutant prM-E gene fragments with NotI-digested ZIKV replicon was directly transfected into BHK-21 cells (Supplemental Figure S3A). After a 120 h incubation, the cultured media of the transfected cells were harvested as the first passage (P1) of wild type and prM-mutant infectious clones (Supplemental Figure S2). The second, and third passages (P2, and P3) of ZIKV wild type and prM-mutant i.c.s. were collected from the cultured media of BHK-21 cells infected with P1 and P2 i.c. after 120 h of incubation, respectively. Relative CPE and EGFP reporters in mock and infected cells were examined inspected 120 h post infection. In addition, the expression of ZIKV proteins in mock and infected cells was evaluated using immunofluorescence staining with primary antibodies against E and NS5 proteins (GeneTex, Inc.) plus anti-rabbit IgG conjugated with Alexa 555 (A-21428; Invitrogen, ThermoFisher Scientific, Waltham, MA, USA). The yield and antigenicity of WT, N139S, and E143K P3 i.c.s in the cultured media of P2 i.c.-infected cells were assessed using a TCID50 assay in BHK-21 cells and Western blotting with primary anti-E and anti-prM antibodies. The TCID50 and Western blotting assays were accomplished, as mentioned above.

### 2.4. Infectivity and Cytopathicity of Wild Type and PrM-Mutant SRIPs and Infectious Clones of ZIKV Natal RGN

In the assays with ZIKV SRIPs, prM-WT/E, prM-N139S/E, and prM-E143K/E expressing TE671 or SF268 cells were infected with indicated SRIPs at an MOI of 0.5. Relative levels of CPE and EGFP reporters in mock and SRIP-infected cells were examined 72 and 120 h post infection using a light microscopy for CPE, and a fluorescence microscopy for the EGFP reporter, respectively. In addition, relative fluorescence intensity of EGFP reporter in infected cells was measured at an excitation wavelength of 485 nm and an emission wavelength of 535 nm using a SpectraMax^®^ iD3 Multi-Mode Microplate Reader (Molecular Devices), and then normalized by the background fluorescence of the mock-infected cells. Meanwhile, the cell survival rate of SRIP-infected cells was explored after 120 h of infection using Cytoscan™ LDH Cytotoxicity Assay (G-Biosciences). Meanwhile, mock- and SRIP-infected cells were harvested 120 h post infection, stained using the propidium iodide (PI) solution, and then analyzed by flow cytometry, as described in our prior report [19]. In addition, the caspase activities in SRIP-infected cells were investigated using Western blotting assay. The lysate from SRIP-infected cells mixed with the Loading Dye was run in 10% SDS-PAGE, and then electrophoretically transferred onto the nitrocellulose membrane with immune-reactive bands, which were reacted with primary antibodies against caspases 1 (Cell Signalling, Danvers, MA, USA), 3 (Calbiocem), and 4 (Cell Signalling), β-actin (Cell Signalling), and HRP-conjugated anti-mouse or anti-rabbit IgG antibodies (Invitrogen, Carlsbad, CA, USA). Finally, the bands were imaged by MultiGel-21 after being developed by ECLTM Western Blotting Detection Reagents. In the assays with ZIKV i.c., TE671, SF268, and HMC3 cells were infected with WT, N139S, and E143K i.c. at an MOI of 0.5, respectively. Relative levels of CPE and EGFP reporter in i.c.-infected cells were examined 48 and 72 h post infection using a light microscopy for CPE, a fluorescence microscopy for EGFP reporter, and SpectraMax^®^ iD3 Multi-Mode Microplate Reader for the fluorescence intensity of EGFP reporter in mock- and i.c.-infected cells. Meanwhile, the virus yields in the cultured media of i.c.-infected cells harvested 72 h post infection were quantified by a TCID50 assay in BHK-21 cells. To detect the relative levels of viral RNA genome in i.c.-infected cells, the total RNAs of TE671 and SF268 cells infected with indicated ZIKV i.c.s at an MOI of 0.5 were extracted 72 h post incubation using the PureLink Mini Total RNA Purification Kit (Thermo Fisher Scientific, Waltham, MA, USA). After reverse transcription of RNAs into cDNA, relative viral RNA genome levels were measured using SYBR Green-based real-time PCR with ZIKV E-specific primer pairs 5′-CAAGATCCCGGCTGAAACACTG-3′ and 5′-TTCTCCCCGACTCCTATGACAATG-3′. Finally, relative viral RNA genome levels in i.c.-infected cells were normalized to glyceraldehyde 3-phosphate dehydrogenase (GAPDH), according to our prior report [17].

### 2.5. Time-of-Addition/Removal Assays with Wild Type and PrM-Mutant Infectious Clones of ZIKV Natal RGN

The time-of-addition/removal assays included attachment and entry modes. In the attachment mode, the monolayers of TE671 and SF268 cells pre-chilled at 4 °C for 1 h were incubated with WT, N139S, and E143K i.c.s (MOI = 1) at 4 °C for an additional 1 h, washed by cold phosphate-buffered saline (PBS), cultured for 18 h at 37 °C, and then stained using IFA with anti-ZIKV E protein plus anti-rabbit IgG conjugated with Alexa 555 and DAPI (4′,6-diamidino-2-phenylindole). Finally, relative attachment activity was measured by the ratio of E-positive cells to total nuclei, according to the image analysis of stained cells using SparkMaster program with Image J software. In the entry mode, the cells were infected with indicated ZIKV Natal RGN i.c.s (MOI = 1) for 1 h at 37 °C, washed by PBS, cultured for 18 h at 37 °C, and then stained using IFA and DAPI. Finally, relative entry activity of WT, N139S, and E143K i.c.s was measured by the ratio of E-positive cells to total cells.

### 2.6. Assays of Extracellular and Intracellular Virion Production in the Cells Infected by Wild Type and PrM-Mutant Infectious Clones of ZIKV Natal RGN

TE671 and SF268 cells were infected with WT, N139S, and E143K i.c.s (MOI = 1) for 1 h at 37 °C, washed by PBS, and added fresh culture media for an additional 48 h of incubation at 37 °C. Finally, cultured media containing extracellular virions and i.c.-infected cells covering intracellular virions were collected, in which the serial dilution of cultured media and the lysate of infected cells through three freeze–thaw cycles were applied to evaluate the virus titers using a TICD50 assay in BHK-21 cells.

### 2.7. Co-Localization of ZIKV E Protein and KDEL Receptor 1 in ZIKV Natal RGN i.c.-Infected Cells

To determine the localization of ZIKV E protein and KDEL receptor 1 in infected cells, HMC3 cells were infected with WT, and E143K Natal RGN i.c.s (MOI = 0.5) °C for 48 h at 37 °C and then stained using triple immunofluorescence labeling with rabbit anti-ZIKV E protein plus goat anti-rabbit IgG conjugated with Alexa Fluor 488 (Thermo Fisher Scientific), mouse anti-KDELR1 (Novus Biologicals, Littleton, CO, USA) plus goat anti-mouse IgG conjugated with Alexa Fluor 546, and DAPI (4′,6-diamidino-2-phenylindole). Finally, images of stained cells were recorded using *Olympus DP70* digital color camera system; co-localization of ZIKV E protein, KDELR1, and the nuclei were computed using the DP controller software (Olympus).

### 2.8. Statistical Analysis

Three independent experiments for each assay were performed, for which data were further compared by one-way analysis of variance and Scheffe’s post hoc test using SPSS 12.0 (SPSS, Inc., Chicago, IL, USA). A *p* value of less than 0.05 was considered as a statistically significant result.

## 3. Results

### 3.1. Reverse Mutation at Residues 139 and 143 within the PrM protein of ZIKV Natal RGN SRIPs

**Figure 2:**
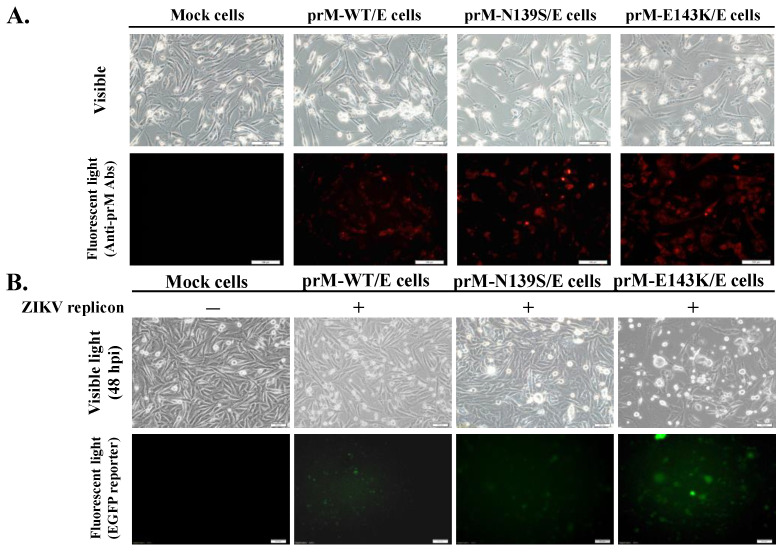

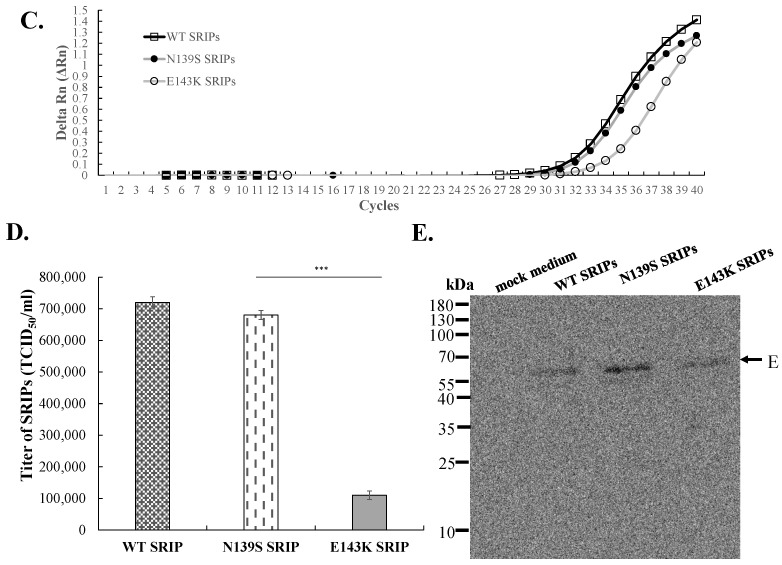
Production of wild type and mutant ZIKV SRIPs by the transfection of cells expressing prM-WT/E, prM-N139S/E, and prM-E143K/E with ZIKV replicon carrying an EGFP reporter. The resultant plasmids were transfected into TE-671 cells, and selected by G148 to establish the cell lines cells expressing prM-WT/E, prM-N139S/E, and prM-E143K/E, respectively (**A**). The expression of prM protein in the cell lines cells was examined using IFA with anti-prM antibodies. Wild type and mutant prM-E expressing cell lines were further transfected with ZIKV Natal RGN replicon. CPE and EGFP reporter in mock cells and replicon-transfected cells were photographed using light and fluorescence microscopy at 48 h post-incubation (**B**). Relative viral genome levels, infectious titers, and antigenicity of WT, N139S, and E143K SRIPs collected from the supernatant of replicon-transfected cell lines were analyzed using real-time RT-PCR with NS5-specific primer pairs (**C**), TCID50 assay in indicated prM/E-expressing cells (**D**), and Western blotting with E-specific antibodies (**E**), respectively. Scale bar, 100 μm. ***, *p* < 0.001.

Initially, single reverse mutations of N139S and E143K within the prM protein were cloned into pcDNA3.1-HisC using PCR products that were amplified by PCR with the mutagenic primers and template of the plasmid containing ZIKV Natal RGN prM-E gene (Supplemental Figure S1). Subsequently, transfected TE671 cells with resultant plasmids sequenced were selected by G418, in which the expression of prM protein was discovered using IFA staining with anti-ZIKV prM antibodies (Figure 2A). Next, prM-WT/E-, prM-N139S/E-, and prM-E143K/E-expressing cells were treated with a second transfection of ZIKV Natal RGN replicon containing an EGFP reporter gene. After a 2-day incubation, detectable levels of CPE and EGFP reporter were remarked in replicon-transfected prM/E-expressing cells. Distinctly, replicon-transfected prM-E143K/E-expressing cells displayed the relatively higher level of CPE and the stronger fluorescent signal of EGFP reporter compared to prM-WT/E and prM-N139S/E-expressing cells transfected with ZIKV Natal RGN replicon (Figure 2B). Lastly, WT, and N139S SRIPs were collected from the cultured media of indicated replicon-transfected cells 3 days post replicon transfection, but E143K SRIPs were harvested 2 days post replicon transfection due to the high rate of cell lysis in prM-E143K-expressing cells transfected with ZIKV Natal RGN replicon. For further assays with equal titers of each SRIPs, wild type and prM-mutant ZIKV Natal RGN SRIPs were quantified using real-time RT-PCR and TCID50 assays (Figure 2C,D). Real-time RT-PCR with ZIKV NS5-specific primers indicated that the Ct value for E143K SRIPs was higher than WT and N139S SRIPs. Meanwhile, a TCID50 viral titration assay showed that the virus titers of WT, N139S, and E143K SRIPs were 7.2 × 10^5^ TCID_50_/mL, 6.8 × 10^5^ TCID_50_/mL, and 1.1 × 10^5^ TCID_50_/mL, respectively. In addition, Western blotting with primary antibodies against ZIKV E protein also revealed that the immunoreactive signal of approximately 58-kDa band for E143K SRIPs was lower than M-WT and N139S SRIPs (Figure 2E), which was consistent with the result of low titers for E143K SRIPs due to the short period of harvesting time because prM-E143K-expressing cells transfected with ZIKV Natal RGN replicon had a high level of CPE and lysed cells 2 days post replicon-transfection. The phenomenon implies the different biological properties between wild type and prM-mutant SRIPs of ZIKV Natal RGN.

### 3.2. Comparing Infectivity and Cytopathogenic Effect of Wild Type and PrM-Mutant SRIPs of ZIKV Natal RGN

**Figure 3:**
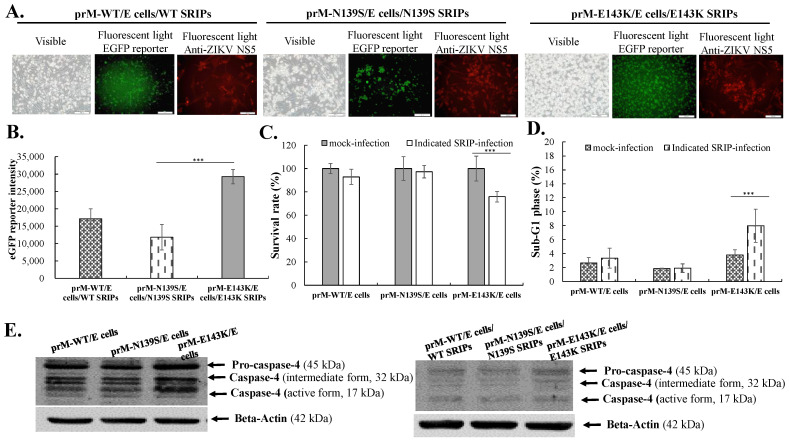
Infectivity and cytopathicity of WT, N139S, and E143K SRIPs of ZIKV Natal RGN in human rhabdomyosarcoma TE671 cells. The cells expressing prM-WT/E, prM-N139S/E, and prM-E143K/E were infected by WT, N139S, and E143K SRIPs at a MOI of 0.5. The appearance of cytopathic change, EGFP reporter signal, and ZIKV NS5 expression in SRIP-infected cells were photographed using light and fluorescence microscopy, as well as IFA staining with anti-NS5 primary antibodies 120 h post-infection (**A**). Relative EGFP fluorescent intensity in the lysate of SRIP-infected cells was measured using a SpectraMax^®^ iD3 Multi-Mode Microplate Reader (**B**). In addition, cell survival rate of SRIP-infected cells was quantitated using LDH assay (**C**). Sub-G1 phase percentage of mock and SRIP-infected cells was examined using flow cytometry after PI staining (**D**). Relative protein levels of pro- and cleaved forms of caspase 4 were analyzed using Western blotting with caspase 4 specific antibodies, respectively (**E**). Scale bar, 100 μm. ***, *p* < 0.001.

**Figure 4:**
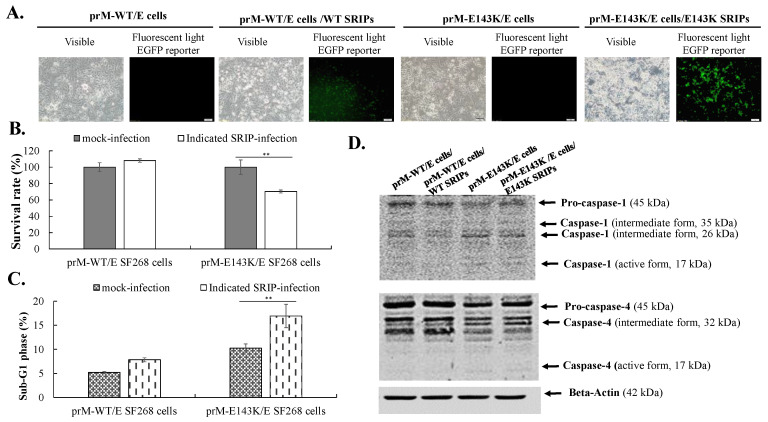
Infectivity and cytopathicity of WT, and E143K SRIPs in human glioblastoma SF268 cells. The cells expressing prM-WT/E, and prM-E143K/E were infected by WT, and E143K SRIPs at a MOI of 0.5. The cytopathic effect and EGFP reporter in mock- and SRIP-infected cells were photographed using light and fluorescence microscopy 120 h post-infection (**A**). Cell survival rate of SRIP-infected cells was quantitated using LDH assay (**B**). Sub-G1 phase percentage of mock and SRIP-infected cells was examined using flow cytometry after PI staining (**C**). Relative protein levels of pro- and cleaved forms of caspases 1/4 were analyzed using Western blotting with caspases 1/4 specific antibodies, respectively (**D**). Scale bar, 100 μm. **, *p* < 0.01.

To examine the relative infectivity and cytopathicity of prM-mutant SRIPs of ZIKV Natal RGN in direct comparison to wild type SRIPs in vitro, TE671 and SF268 cells expressing prM/E proteins were infected with WT, N139S, and E143K SRIPs at an MOI of 0.5, respectively, and cultured for 120 h, in which relative levels of infectivity and cytopathicity were determined (Figure 3 and Figure 4). Wild type and prM-mutant N139S, and E143K SRIPs competently infected prM/E-expressing TE671 and SF268 cells, resulting in the appearance of virus-induced CPE and EGFP reporter signals in infected cells (Figure 3A,B and Figure 4A). In addition, IFA staining with anti-ZIKV NS5 antibodies revealed the expression of ZIKV proteins in infected cells with wild type and pr m mutant SRIPs. Major differences among infected cells showed that E143K SRIP-infected cells had relatively higher levels of virus-induced CPE and EGFP reporter signals compared to the cells infected with WT and N139S SRIPs. Meanwhile, E143K SRIP-infection caused substantial increases in cell death and sub-G1 phase in TE671 and SF268 cells by comparison with WT and N139S SRIP-infection, which had no significant alternation of cell death and sub-G1 phase in infected cells (Figure 3C,D and Figure 4B,C). Additionally, the Western blot analysis of caspase cleavage demonstrated that infection by E143K SRIP, but not WT or N139S SRIPs, induced the activation of caspase 4, not caspases 1 and 3 in TE671 and SF268 cells (Figure 3E and Figure 4D). Notably, a single reverse mutation E143K within the prM protein revolutionized the phenotype of ZIKV Natal RGN SRIPs, with obvious impacts on infective and cytopathogenic activities.

### 3.3. Understanding the Replication Cycle of ZIKV Natal RGN E143K Infectious Clone

**Figure 5:**
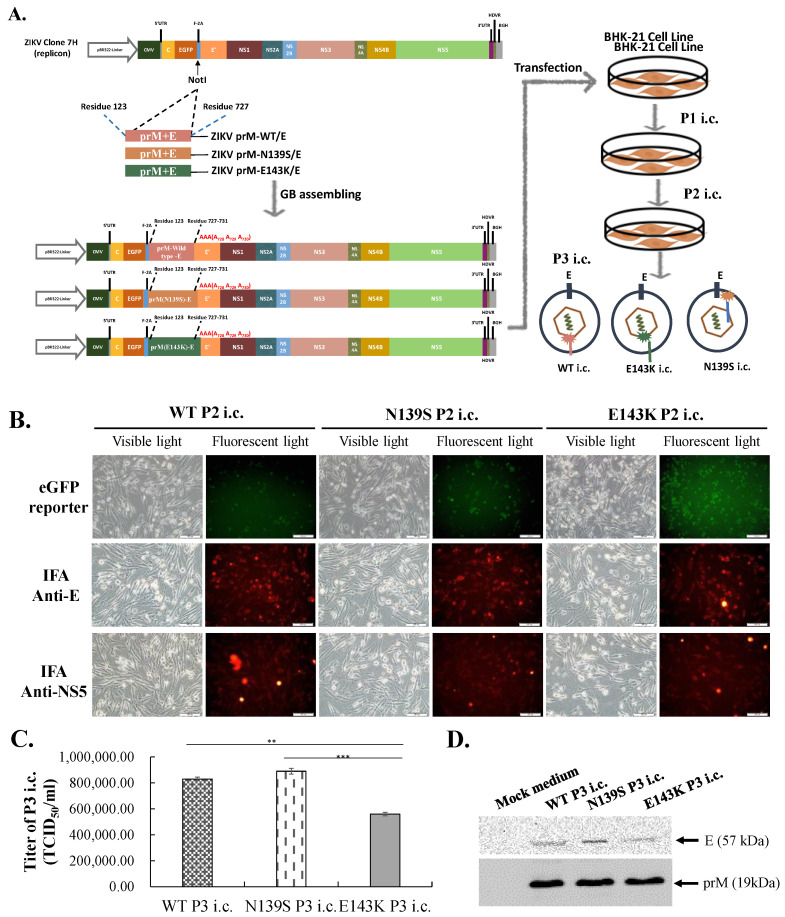
Production, antigenicity, and quantitation of wild type and prM-mutant P3 infectious clones (i.c.s) of ZIKV Natal RGN. Schematic representation for the generation of wild type and prM-mutant infectious clones (i.c.s) of ZIKV Natal RGN (**A**). NotI site between FMDV-2A (F-2A) and E’ (from ZIKV Nucleotide 2307) of ZIKV Natal RGN was the unique restriction enzyme site for the digestion. NotI-digested ZIKV replicon was in-frame assembled with the prM-E fragment (Nucleotides 474–2297) and amplified by PCR with the specific primer pair listed in Supplemental Figure S2B using Gibson Assembly reaction, which was then transfected into BHK-21 cells. The cultured media of transfected cells that were re-infected twice in BHK-21 cells were collected to test the biological properties of WT, N139S, and E143K i.c.s of ZIKV Natal RGN. BHK-21 cells were infected with P2 i.c.s for 120 h; CPE, EGFP reporter, and ZIKV proteins in transfected or infected cells were examined using light/fluorescent microscopy and IFA with anti-E and anti-NS5 antibodies (**B**). The cultured media of infected cells collected as the third passage (P3) of WT, N139S, and E143K i.c.s, for which the virus titer of each P3 i.c. in the media was determined using TCID50 assay in BHK-21 cells (**C**). The antigenicity of each P3 i.c. was analyzed using Western blotting with anti-E and anti-prM antibodies (**D**). Scale bar, 100 μm. **, *p* < 0.01; ***, *p* < 0.001.

**Figure 6:**
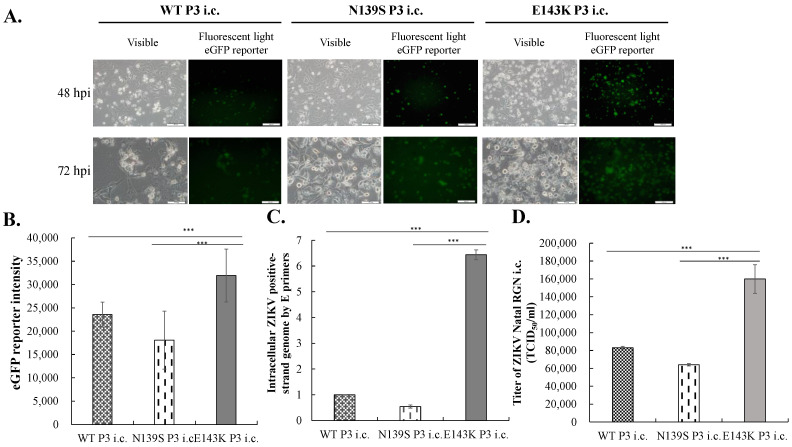
Infectivity and replication efficiency of WT, N139S, and E143K P3 i.c.s of ZIKV Natal RGN in TE671 cells. The cells were infected with prM-WT, prM-N139S, and prM-E143K i.c.s at a MOI of 0.5. The cytopathic effect and EGFP reporter in i.c.-infected cells were photographed using light and fluorescence microscopy 72 h post-infection (**A**). Relative EGFP fluorescent intensity in the lysate of infected cells was measured using a SpectraMax^®^ iD3 Multi-Mode Microplate Reader (**B**). In addition, total RNAs in infected cells were extracted using PurLink RNA Mini Kit, and then performed using RT-PCR with gene-specific primer pairs. Relative RNA levels of positive-sense ZIKV genome in i.c.-infected cells were normalized by GAPDH mRNAs (**C**). The virus titer in each cultured medium from i.c.-infected cells was determined using TCID50 assay (**D**). Scale bar, 100 μm. ***, *p* < 0.005.

**Figure 7:**
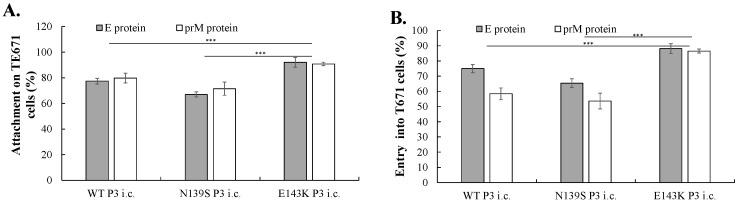

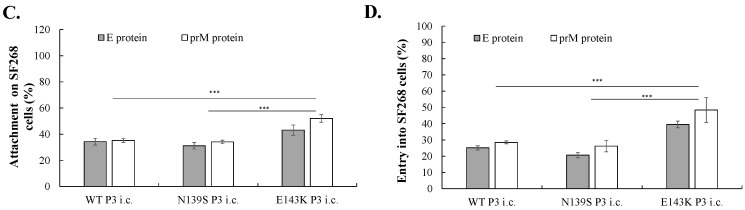
A comparison of virus attachment and cell entry of WT, N139S, and E143K i.c.s in TE617 (**A**,**B**) and SF268 (**C**,**D**). In the attachment mode, the cells were pre-incubated at 4 °C for 1 h, and the media were removed; the ZIKV P3 i.c. was infected at an MOI of 0.5 for 1 h, washed by cold PBS, and incubated with the fresh culture media. After an 18 h incubation, the cell monolayer was performed by IFA with anti-E and anti-prM antibodies and DAPI staining. Virus attachment activity of indicated ZIKV P3 i.c.s was determined according to the percentage of ZIKV E-positive cells in total cells (**A**,**C**). In the entry mode, the cells were infected by ZIKV P3 i.c.s at an MOI of 0.5 for 1 h, washed by PBS, incubated with the fresh culture media for 18 h, and then implemented by IFA and DAPI staining. Cell entry activity of indicated ZIKV i.c. was determined according to the percentage of ZIKV E-positive cells (**B**,**D**). ***, *p* value < 0.001.

**Figure 8:**
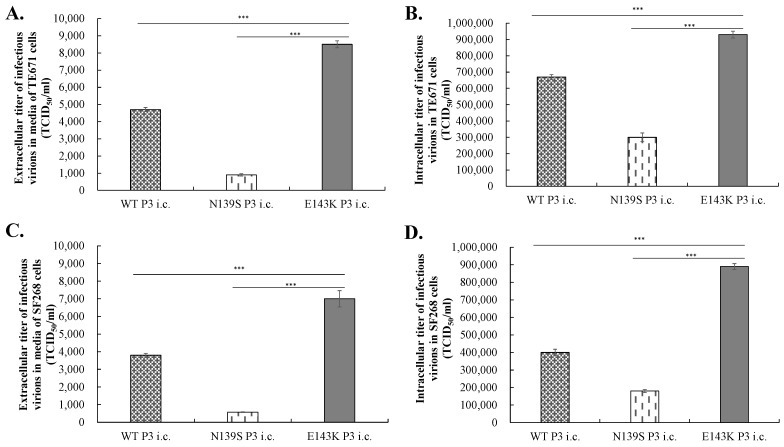
Intracellular and extracellular virion productions of ZIKV Natal RGN WT, N139S, and E143K i.c.s in TE617 (**A**,**B**) and SF268 (**C**,**D**). The cell monolayer was infected with WT, N139S, and E143K P3 i.c.s (MOI = 0.5) for 1 h, washed with PBS, and incubated with fresh media. After an additional 48 h incubation, the cultured media containing extracellular virions from indicated i.c.-infected cells were collected (**A**,**C**). Meanwhile, the monolayer of infected cells containing intracellular virions were washed, harvested, and then lysed by three freeze–thaw cycles (**B**,**D**). The virus titer in the cultured media (extracellular virions) and cell lysate supernatants (intracellular virions) were determined by TCID_50_ assay. ***, *p*-value < 0.001. Scale bar, 100 μm.

Compared with the infectivity of ZIKV Natal RGN SRIPs, wild type and prM-mutant infectious clones (i.c.s) were further generated to characterize viral RNA and protein synthesis, virus yield, and replication kinetics in TE671 and SF268 cells (Figure 5, Figure 6, Figure 7 and Figure 8 and Supplemental Figures S2–S4). Initially, WT, N139S, and E143K i.c. were collected from the media of BHK-21 cells transfected with the Gibson Assembly reaction of indicated prM-E gene fragments with NotI-digested ZIKV Natal RGN replicon, and passaged thrice in BHK-21 cells (designated P1, P2, and P3 i.c.s) (Figure 5, and Supplemental Figures S2 and S3). The expression of EGFP and ZIKV proteins was discovered in the transfected cells with the Gibson Assembly reaction and the infected cells with P1, P2, and P3 i.c.s of wild type and prM-mutant ZIKV Natal RGN. Furthermore, a TCID50 assay in BHK-21 cells ascertained that the infectious titers of P3 ZIKV Natal RGN i.c. were 8.3 × 10^5^ TCID_50_/mL for WT i.c., 8.9 × 10^5^ TCID_50_/mL for N139S i.c., and 5.6 × 10^5^ TCID_50_/mL for E134K i.c., respectively (Figure 5C). Additionally, the antigenic reactivity of WT, N139S, and E143K P3 i.c.s was characterized using Western blotting with primary antibodies against ZIKV E and prM proteins, respectively (Figure 5D). The results reveal that WT, N139S, and E143K i.c. of ZIKV Natal RGN were generated using the in vitro ligation of prM-E gene fragment with ZIKV replicon by the Gibson assembly method.

Next, replication kinetics and virus yields of WT, N139S, and E143K i.c. in TE671 and SF268 cells were evaluated (Figure 6 and Supplemental Figure S4). Cells were infected with WT, N139S, and E143K P3 i.c.s (MOI = 0.5), respectively. The E143K P3 i.c. caused relatively higher levels of cytopathic effects and EGFP signals in infected cells than WT and N139S P3 i.c.s (Figure 6A,B, Supplemental Figure S4A,B). Moreover, E143K P3 i.c. had a more efficient release of ZIKV particles and higher viral RNA synthesis in SF268 and TE671 cells compared to WT and N139S P3 i.c.s. The results suggest that E143K i.c. replication was significantly more efficient compared to WT and N139S i.c.s in vitro.

### 3.4. Identifying the Mechanism for the Efficient Infection of E143K Natal RGN i.c.

To adequately assess the differences in entry events and virion egress between the wild type and prM-mutant i.c.s of ZIKV Natal RGN, attachment and entry/fusion assays were performed by one-hour incubation of WT, N139S, and E143K P3 i.c.s (MOI = 1) with TE671 and SF268 cells at 4 °C and 37 °C, respectively, and an additional 18 h incubation at 37 °C after washing with PBS to remove un-bound viruses. WT, N139S, and E143K P3 i.c.s had the greater activity of attachment and entry in TE671 cells compared to SF268 cells. Notably, E143K P3 i.c. exhibited significantly higher activities of attachment and viral entry in both types of cells compared to WT and N139S P3 i.c.s (Figure 7). Subsequently, the TCID50 assay in BHK-21 cells was used to measure the titers of intracellular and extracellular infectious viruses for examining the maturation and release of WT, N139S, and E143K P3 i.c.s in TE671 and SF268 cells 48 h post infection (Figure 8). Like the results for attachment and entry assays, WT, N139S, and E143K P3 i.c.s exhibited higher titers of intracellular and extracellular virions in TE671 cells compared to SF268 cells. Markedly, E143K P3 i.c. and N139S P3 i.c. had higher titers of intracellular and extracellular virions than WT P3 i.c., from 1.4- to 2.2-fold, and from 3.1- to 12.8-fold, respectively (Figure 8). Taken together, these results demonstrate that the entry event and virion egress of the E143K i.c.s were superiorly efficient compared to the WT and N139S i.c.s of ZIKV Natal RGN in TE671 and SF268 cells.

**Figure 9:**
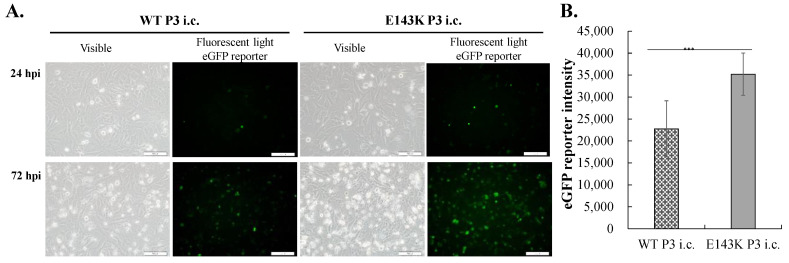

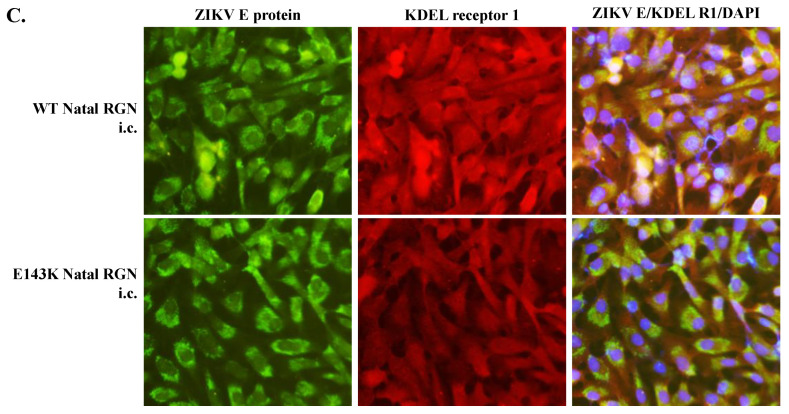
The effect of the reverse mutation E143K within the pr region of ZIKV Nata RGN prM protein on the intracellular localization of ZIKV E protein and KDEL receptor 1. The relative level of CPE and EGFP signal in infected cells with WT and E143K Nata RGN i.c.s was examined using optical/fluorescence microscopy (**A**) and a SpectraMax^®^ iD3 Multi-Mode Microplate Reader (**B**). Moreover, the intracellular localization of ZIKV E protein and KDEL receptor 1 was detected using triple immunofluorescence labeling with rabbit anti-ZIKV E protein bound to secondary antibodies conjugated with Alexa Fluor 488, mouse anti-KDELR1 probed by secondary antibodies conjugated with Alexa Fluor 546, and DAPI (**C**). ***, *p*-value < 0.001.

Since the interaction of KDEL receptors 1 and 2 (KDELR1 and KDELR2) with the three positively charged amino acids within the pr region of DENV prM was identified as being involved in the translocation of DENV particles from the ER to the Golgi [20], the relationship between the reverse mutation E143K with the pr region of Natal RGN prM, and the localization of ZIKV E protein and KDEL receptor 1 in infected cells was subsequently examined (Figure 9). E143K Natal RGN i.c. infection provoked relatively higher levels of CPE and EGFP signal in HMC3 cells compared to WT Natal RGN i.c. (Figure 9A,B), which was consistent with the results of TE671 and SF268 cells infected by E143K Natal RGN i.c and SRIP in comparison with WT and N139S Natal RGN i.c.s or SRIPs. An image analysis of i.c.-infected HMC3 cells using triple immunofluorescence labeling specified the partial co-localization of E and KDELR1 in HMC3 cells infected with WT, and E143K Natal RGN i.c.s. Vitally, the majority of ZIKV E protein was observed as the aggregated form that was localized near the cell plasma membrane of E143K Natal RGN i.c.-infected cells. In contrast, the greater part of E was detected in the cytoplasm of WT Natal RGN i.c.-infected cells (Figure 9C). The results suggest that the reverse mutation E143K with the pr region of Natal RGN prM affected the efficiency of viral protein transport to the surface of the cell plasma membrane through regulating the secretory pathway.

## 4. Discussion

The study generated wild type and prM-mutant SRIPs and i.c. of ZIKV Natal RGN using reverse genetic approaches (Figure 1 and Figure 5, and Supplemental Figures S1–S3) and then characterized the differences in the virological properties among WT, N139S, and E143K SRIPs, as well as i.c.s of ZIKV Natal RGN. The SRIPs of ZIKV Natal RGN were the first choice for examining the virological phenotype change of N139S and E143K within the pr region of the prM protein due to the high genetic stability of cloned ZIKV prM and E gene fragment, as well as the ZIKV replicon when the plasmids were maintained in bacteria. The cloning process of pBR322 plasmid launched CMV promoter-driven ZIKV i.c. has also been tried in the study, but nonsense mutations and deletion were identified in the constructed clones, which implied that the full-length cDNA of ZIKV Natal RGN genome could be toxic to *E. coli* 10B and other strains. Thus, Gibson Assembly reaction of prM-E gene fragment (PCR product) and NotI-digested ZIKV replicon was directly transfected into TE671 cells to generate the i.c.s of ZIKV Natal RGN. The results indicate that transfection of mammalian cells with Gibson Assembly ligation of full-length viral genome cDNAs is a suitable and alternative approach to generating viral infectious clones.

A reverse mutation E143K, not N139S, within the precursor (pr) region of ZIKV Asian-lineage prM protein replaced as African-lineage strains was associated with an increase in infectivity and cytopathogenicity with relatively higher levels of cytopathic effect, EGFP reporter, viral RNA and protein synthesis, and virus yield in three types of human cell lines (Figure 2, Figure 3, Figure 4, Figure 5, Figure 6 and Figure 9). The results indicate that the E143K SRIP and i.c.s of ZIKV Natal RGN exhibited similar biological characteristics to ZIKV African-lineage and pre-epidemic ZIKV Asian-lineage isolates, which display faster replication kinetics with higher peak virus titers and vRNA synthesis in many vertebrate cells in comparison to epidemic ZIKV Asian-lineage isolates, including ZIKV Natal RGN strain [9,10,11]. Remarkably, the residue Lys143, not Ser139, in the pr region of prM protein was 1 of 10 amino acids conserved in ZIKV African-lineage isolates that were proposed to be linked with the high virulence of ZIKV African-lineage MR766 strain [16]. WNV mutant with amino acid substitution at the positive charge residue Lys31 in the pre region of prM protein showed a lower efficiency of replication kinetics and reduced infectivity compared to wild type parent virus [21]. N139S SRIP and i.c. of ZIKV Natal RGN exhibited less cytotoxic and slow replication kinetics in TE671, SF268 and HMC3 cells, a finding that is consistent with the prior report, in that S139N, but not N139S, within the prM protein of ZIKV Asian-lineage pre-epidemic strains markedly exhibited neurovirulence in neonatal mice and high infectivity to human and mice NPCs [15]. Therefore, the results indicate that the reverse mutation E143K restored the infectivity and cytopathicity in recombinant ZIKV Natal RGN, much like ZIKV African-lineage isolates.

After the furin cleavage of prM in pr m junction, the pr peptides remain bound to the E protein for preventing premature fusion when virions were transported across the acidic trans-Golgi network, followed by the release of pr-attached virions from the infected cells [22]. Markedly, the pr-attached virions and prM incomplete cleaved virions were exhibited as heterogeneous and dynamic infectious particles with a variation in tissue tropism, which increased the wide range of cells susceptible to ZIKV [23]. The modeling of the three-dimensional structure of the prM/E complex showed that the residue Lys143 (or Glu143) in the prM was not located within or near the cleavage site of host furin, for which the reverse mutation E143K was unlikely to disturb the furin cleavage during the virus maturation process. Additionally, the residue 143 was not involved in the interaction of the pr region of prM with the interface between Domains I and II of the E protein based on the crystal structures of DENV pr-E complexes (PDB codes: 3C5X and 3C6E) [24]. Importantly, the reverse mutation E143K within the pr region led to an increase in positive charge and hydrophilicity on the exposed surface of the virions, which might signify positive-charge-based interactions with ZIKV attachment factors (such as heparan sulfate and chondroitin sulfate) and the negatively charged membrane [25]. The claudin-1 protein, one of predicted DENV receptors, exhibited a direct interaction with pr, M and prM proteins, which was essential for the efficient DENV entry [26]. Thus, previous studies revealed that the increase in positive charges on the surfaces of E143K ZIKV Natal RGN i.c.s may account for the relatively higher attachment and entry activities in TE671 and SF268 cells than WT and N139S i.c.s (Figure 7), implying that the pr region of prM might be one of the viral factors involved in virus attachment and entry during ZIKV replication.

The function of the pr region of DENV and JEV prM proteins was demonstrated to modulate the secretion of dengue virus in infected cells, such as binding to class II ADP-ribosylation factors and KDEL receptors [20,27,28]. A K21T substitution in the pr region of the dengue virus prM protein, as the same residue at the same position Lys143 in ZIKV, significantly reduced the interaction with KDEL receptors 1 and 2, which resulted in a decrease in dengue virus egress from the cell [20]. The mutation at Lys31 in the pr region of WNV prM was also demonstrated to result in the accumulation of intracellular prM-E heterodimers in the ER and ER-Golgi intermediary compartments, and then impede the secretion of WNV virus-like particles from the infected cells [21]. The results of previous reports supported our findings, in that the reverse mutation E143K of ZIKV Natal RGN increased in the extracellular virus yield, multiplied the intracellular virus titer, and enhanced the E protein aggregation localized near the cell plasma membrane in the secretory pathway (Figure 8 and Figure 9). This suggested that the reverse mutation E143K in the pr region of prM was associated with an improvement in the efficiency of ZIKV Natal RGN assembly and egress.

## 5. Conclusions

This study demonstrated the virological characteristics of wild type, N139S, and E143K mutants of ZIKV Natal RGN SRIPs and i.c., identified the relatively higher replication efficiency of E143K mutant compared to wild type virus and N139S mutants. Notably, the reverse mutation E143K in the pr region of Natal RGN prM protein, which was replaced as the same residue in ZIKV African lineages, revealed the significance of the increase in infectivity and cytopathicity. The results indicate the positively charged amino acid residue Lys143 that was highly conserved in the pr region of prM of ZIKV African lineages plays a crucial role in viral replication kinetics, including viral attachment, entry, assembly and egress. Thus, lysine replaced by glutamic acid at the residue position 143 in the pr region of prM can alter phenotypes, in particular, those that have a lower replication efficiency of ZIKV Asian-lineage isolates with the attenuation of viral virulence.

## Data Availability

Not applicable.

## Supplementary Materials

The following supporting information can be downloaded at: https://www.mdpi.com/article/10.3390/v14071572/s1, Figure S1: Cloning map (A) and sequencing analysis (B) of wild type and mutant prM-E gene fragments into the mammalian expression vector pcDNA3.1-HisC. The primer pairs used for the PCR reaction to synthesize wild type and mutant prM-E gene fragments are listed in Figure A; Figure S2: In vitro ligation of prM-E gene fragment with ZIKV Natal RGN replicon using Gibson Assembly reaction. ZIKV replicon, for which the cloning map is shown in the top of Figure A, was purified, digested by the restriction enzyme NotI, and then analyzed using 0.8% agarose gel electrophoresis (A). The gene fragments of prM-WT/E, prM-N139S/E, and prM-E143K/E were amplified using PCR with indicated resultant plasmids as the templates and the primer pair listed in Figure B. The PCR products were analyzed in 1% agarose gel electrophoresis (B). Subsequently, NotI-digested ZIKV replicon was in vitro ligated with each PCR product using Gibson Assembly reaction (C); Figure S3: Production of P1 and P2 infectious clones (i.c.s) of wild type and mutant ZIKV Natal RGN. Initially, the mixture from each Gibson Assembly reaction was directly transfected into BHK-21 cells (A), where cultured media were collected as the first passage (P1) of parent wild type and prM mutant infectious clones (A). The second passage (P2) of ZIKV i.c.s was harvested from the cultured media of infected BHK-21 cells with P1 i.c. (B). After a 120-h incubation, CPE, Egfp reporter, and ZIKV proteins in transfected or infected cells were examined using light/fluorescent microscopy and IFA with anti-E and anti-NS5 antibodies. Scale bar, 100 μm; Figure S4: Infectivity and replication efficiency of WT, N139S, and E143K i.c.s of ZIKV Natal RGN in TE671 cells. The cells were infected with WT, N139S, and E143K P3 i.c.s at a MOI of 0.5. The cytopathic effect and eGFP reporter in i.c.-infected cells were photographed using light and fluorescence microscopy 72 h post-infection (A). Relative eGFP fluorescent intensity in the lysate of infected cells was measured using a SpectraMax® iD3 Multi-Mode Microplate Reader (B). Additionally, total RNAs in infected cells were extracted using PurLink RNA Mini Kit, and then performed using RT-PCR with gene-specific primer pairs. Relative RNA levels of positive-sense ZIKV genome in i.c.-infected cells were normalized by GAPDH mRNAs (C). The virus titers in each cultured medium from i.c.-infected cells were determined using TCID50 assay (D). Scale bar, 100 μm.
