# Supplementary material for: A Reverse Mutation E143K within the PrM Protein of Zika Virus Asian Lineage Natal RGN Strain Increases Infectivity and Cytopathicity
Source: Viruses. 2022 Jul 20;14(7):1572. doi: 10.3390/v14071572 (PMC9317194; doi:10.3390/v14071572)
Supplement: Supplementary file 1 [file viruses-14-01572-s001.zip › viruses-1793899-supplementary.pdf]

Supplementary Materials

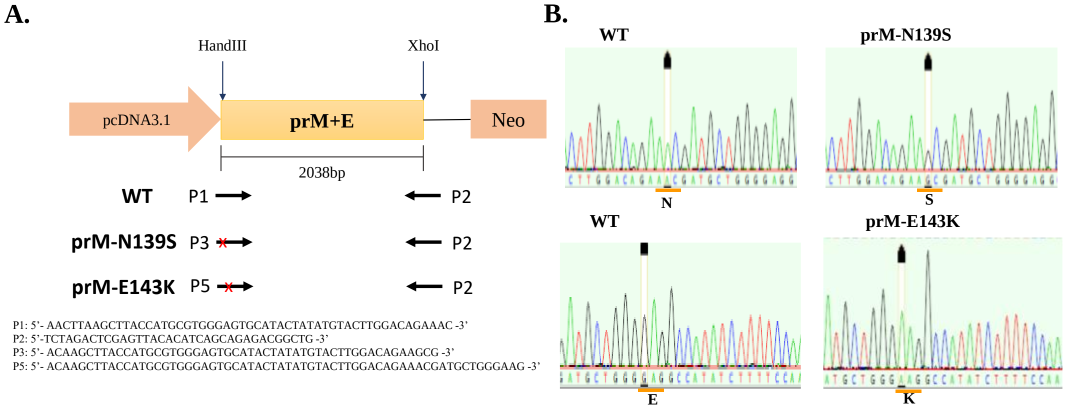

Supplemental Figure S1. Cloning map (A) and sequencing analysis (B) of wild type and mutant prM-E gene fragments into the mammalian expression vector pcDNA3.1-HisC. The primer pairs used for the PCR reaction to synthesize wild type and mutant prM-E gene fragments were listed in Figure A.

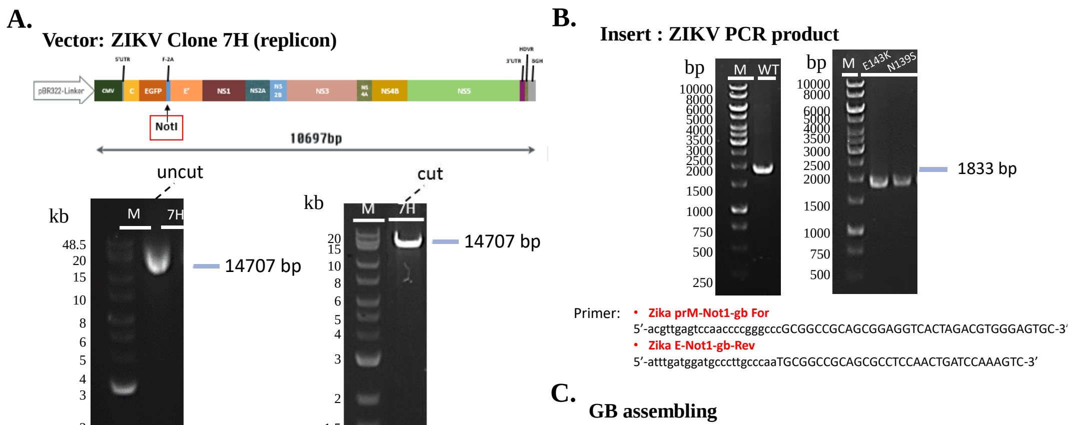

Recommended DNA Molar Ratio	vector : Insert = 1:2
Total Amount of Fragments	Vector：ZIKA clone 7H= 1.9 μl<br>Insert：WT,N139S,E143K,P287L= 3.8 μl
NEBuilder HiFi DNA Assembly Master Mix	10 μL
ddH2O	4.3 μL
Total Volume	20 μL

Supplemental Figure S2. In vitro ligation of prM-E gene fragment with ZIKV Natal RGN replicon using Gibson Assembly reaction. ZIKV replicon, which was shown in the top of Figure A, was purified, digested by the restriction enzyme NotI, and then analyzed using 0.8% agarose gel electrophoresis (A). The gene fragments of prM-WT/E, prM-N139S/E, and prM-E143K/E were amplified using PCR with indicated resultant plasmids as the templates and the primer pair listed in Figure B. The PCR products were analyzed 1% agarose gel electrophoresis (B). Subsequently, NotI-digested ZIKV replicon was in vitro ligated with each PCR product using Gibson Assembly reaction (C).

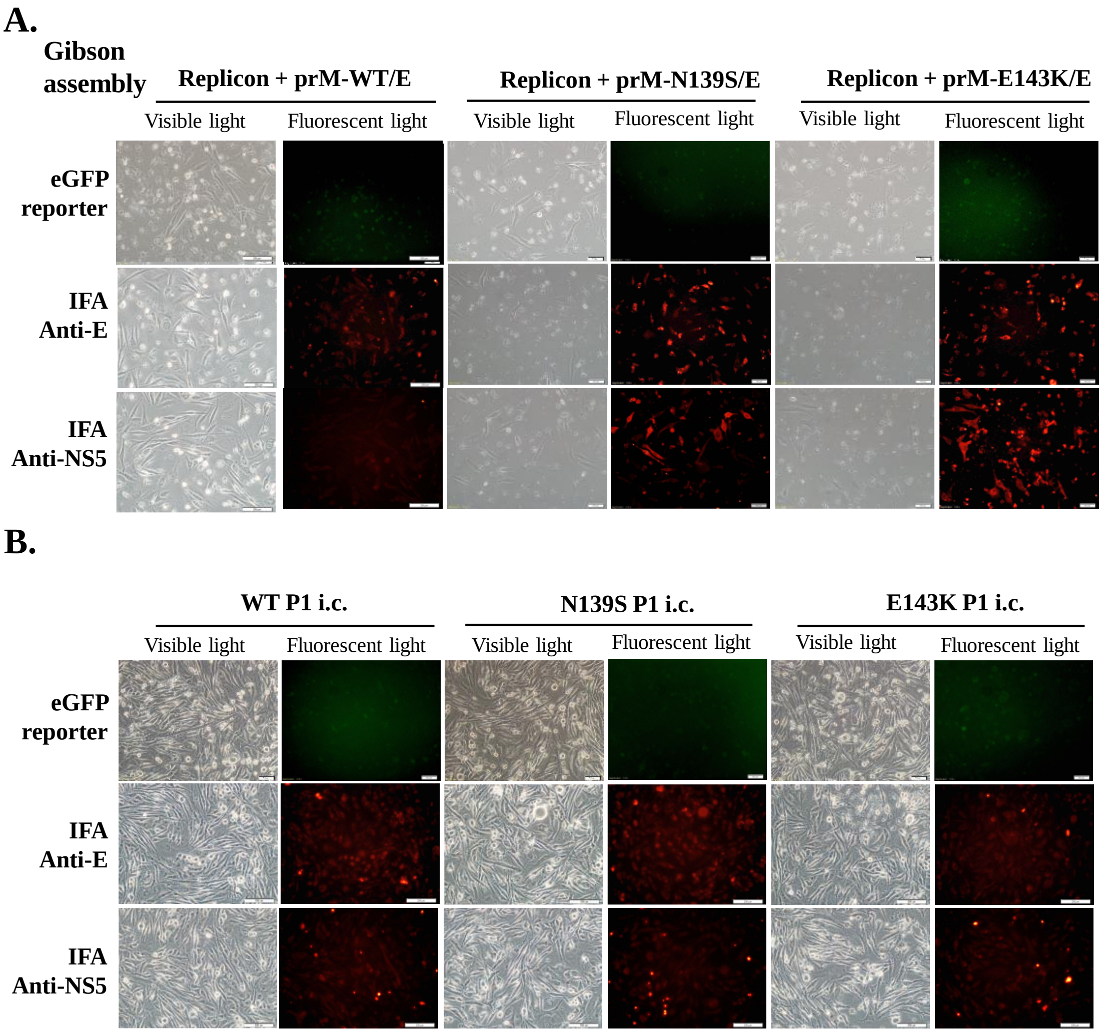

Supplemental Figure S3. Production of P1 and P2 infectious clones (i.c.s) of wild type and mutant ZIKV Natal RGN. Initially, the mixture from each Gibson reaction was directly transfected into BHK-21 cells (A), which cultured media were collected as the first passage (P1) of parent wild type and prM mutant infectious clones (A). The second passage (P2) of ZIKV i.c.s was harvested from the cultured media of infected BHK-21 cells with P1 i.c. (B). After a 120-hour incubation, CPE, eGFP reporter, and ZIKV proteins in transfected or infected cells were examined using light/fluorescent microscopy and IFA with anti-E and anti-NS5 antibodies. Scale bar, 100 µm.

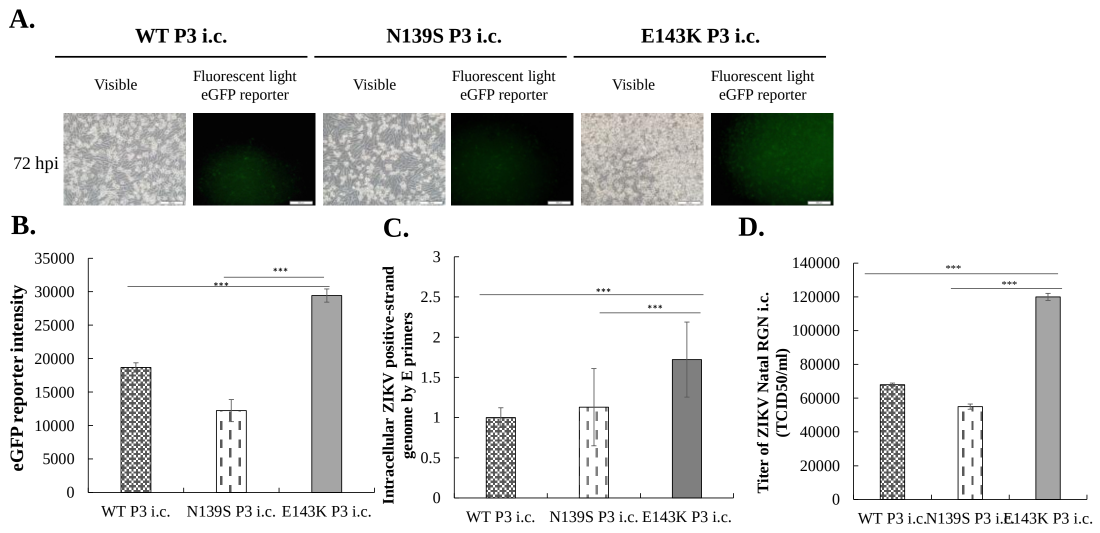

Supplemental Figure S4. Infectivity and replication efficiency of WT, N139S, and E143K i.c.s of ZIKV Natal RGN in TE671 cells. The cells were infected with WT, N139S, and E143K P3 i.c.s at a MOI of 0.5. The cytopathic effect and eGFP reporter in i.c.-infected cells were photographed using light and fluorescence microscopy 72 h post-infection (A). Relative eGFP fluorescent intensity in the lysate of infected cells was measured using a SpectraMax® iD3 Multi-Mode Microplate Reader (B). Additionally, total RNAs in infected cells were extracted using PurLink RNA Mini Kit, and then performed using RT-PCR with gene-specific primer pairs. Relative RNA levels of positive sense ZIKV genome in i.c.-infected cells was normalized by GAPDH mRNAs (C). The virus titer in each cultured medium from i.c.-infected cells was determined using TCID50 assay (D). Scale bar, 100 µm.
